# The Switch in the Diagnosis of Mitochondrial Diseases from the Classical ‘Function First’ To the NGS-based ‘Genetics First’ Diagnostic Era

**DOI:** 10.34763/jmotherandchild.20202402si.2005.000008

**Published:** 2020-10-02

**Authors:** Wolfgang Sperl, Saskia Wortmann, René G. Feichtinger, Johannes A. Mayr

**Affiliations:** 1 Department of Pediatrics, University Hospital Salzburg, Paracelsus Medical University Salzburg, Salzburg, Austria

**Keywords:** mitochondrial diseases, OXPHOS, paediatrics, diagnostics, next generation sequencing, exome sequencing, whole genome sequencing, mitochondrial DNA, diagnostic centres

## Abstract

The knowledge of causes and pathophysiology of mitochondrial diseases has increased exponentially in the last four decades. Recently, due to the decreased costs of new sequencing technologies (exome and whole genome sequencing), these technologies were applied more and more in clinical routine. The traditional diagnostic approach (‘biopsy first’) of evaluating the patient and his body fluids and the analysis of enzymes of the oxidative phosphorylation system in skeletal muscle with subsequent Sanger sequencing of single candidate genes (‘from function to gene’) were replaced by next generation sequencing techniques with a diagnostic yield of >40%. In this ‘genetics first’ approach, the detection of new candidate genes necessitates often functional evaluations (‘from gene to function’) leading to reverse phenotyping of affected individuals. The new genetic era has offered a clear new challenge for the responsibility of the diagnostic centres: the interplay of clinicians, geneticists and functional biochemists is a prerequisite for a validated diagnosis. It becomes evident that expanded diagnostics builds an interface to research. Only competence centres with high numbers of patients, clinical and diagnostic experience and exchange of knowledge with other comparable units can fulfil all those requirements.

## Introduction

**Figure 1:**
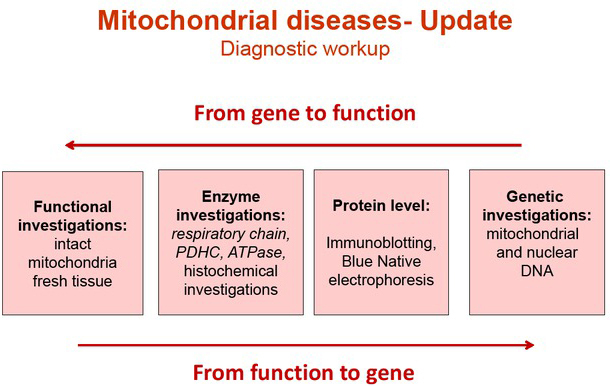
Switch from the classical diagnostic approach ‘function first’ to NGS-based ‘genetics first’

During the last four decades, there was an enormous and continuous increase of knowledge in the molecular basis of mitochondrial diseases. In this overview, we describe the different steps in the development of the diagnosis of mitochondrial diseases. Functional testing was pivotal in the diagnostic approach of the initial period ‘function first’, whereas the availability of next generation sequencing (NGS) from around 2010 onwards allowed a paradigmatic change to ‘genetics first’ (Figure 1). In this overview, we give examples from both eras. Nevertheless, we also demonstrate that the combination of clinical aspects, biochemical investigations and genetic analysis is still important, although now in a different order.

## Enzymatic Decade

In the first era, the late 1970s and 1980s, enzyme defects of the respiratory chain and the pyruvate dehydrogenase complex (PDHC) have been described in children as well as in adults, mostly named as mitochondrial myopathies (1, 2). This was the period of the stepwise elucidation of defects at the enzyme level in the whole pyruvate oxidation pathway (PDHC, Krebs cycle and respiratory chain). In this first period of the description of mitochondrial diseases, muscle and skin biopsies and consecutively enzyme investigations in these tissues were standard as well as a meticulous description of the histology and morphological findings of mitochondria mostly in skeletal muscle. Finally, the decoding of the human mitochondrial DNA (mtDNA) sequence by Andersen in 1981 was the basis for the next step in diagnostics, the genetic elucidation of mitochondrial diseases (3).

## mtDNA Decade

Without any doubt, the first description of mtDNA mutations causing mitochondrial diseases by Anita Harding for mitochondrial DNA deletions in Kearns Sayre syndrome (4) and Douglas Wallace for mtDNA point mutations in Leber hereditary optical neuropathy (5) is a landmark in the diagnostics of mitochondrial diseases. In the second decade, in the 1990s, an increasing number of various mtDNA mutations were detected in the small and beautiful and well-organised DNA molecule. Nevertheless, it turned out soon, as the mtDNA encodes just for 13 structural proteins, 22 tRNAs and 2 rRNAs and therefore the minority of the respiratory chain subunit polypeptides, that mtDNA mutations are only in a minority of patients, especially in paediatrics, responsible for mitochondrial diseases. Concomitantly, a great number of nuclear encoded defects have been described in the following.

Due to the fascinating dual control of the oxidative phosphorylation system (OXPHOS) by two genomes, the possibility of levels of dysfunction is huge. Not only the defects of structural proteins of the OXPHOS system could be affected but also the complex system of the mitochondrial protein, import, processing and aggregation of nuclear encoded mitochondrial proteins as well as the mitochondrially encoded protein synthesis machinery. Mitochondrial dysfunction can also result in a disturbed mtDNA maintenance, a defective lipid membrane and a disturbed motility of the organelle. Furthermore, defects in numerous enzyme cofactors and their underlying metabolism were identified. Therefore, the majority of defects in mitochondrial diseases has turned out to be located in the nuclear genome.

## Function First: Nuclear DNA Mutations (‘from Function to Gene’)

**Figure 2:**
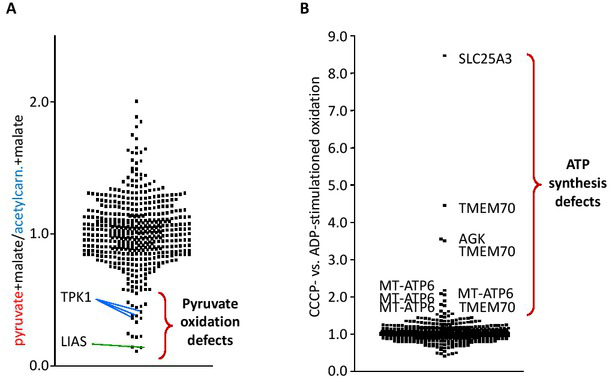
A functional investigation by substrate oxidation analysis of fresh muscle biopsies reveals defects of (A) the pyruvate oxidation route and (B) ATP synthesis

In the third decade, till at least 2010, the standard procedure in the diagnosis of a disorder in the mitochondrial energy metabolism was the performance of a muscle biopsy, measurement of enzymes and finally the confirmation of the diagnosis by genetic testing of candidate genes in known pathways (e.g. subunits of complex in case of a decreased complex I activity). In this period, due to the functional testing of the pyruvate oxidation route in isolated mitochondria, new defects have been detected, especially on the two ends of the pyruvate oxidation route, first in the pyruvate oxidation system itself and second in the ATP synthesis machinery. By functional narrowing down the suspected level of dysfunction, gene defects were suspected in a couple of known candidates and finally proven by Sanger sequencing of one candidate after the other. This classical approach of the strategically logic way from function to a suspected gene could be demonstrated by our group in several diseases in the *pyruvate oxidation route* (Figure 2A) and *ATP synthesis* (Figure 2B) using the functional investigation of intact mitochondria from unfrozen biopsy tissue.

The pyruvate oxidation route starts from pyruvate, the end product of glycolysis, and includes its import into mitochondria and the oxidative decarboxylation by the PDHC to acetylcoenzyme A, which is further metabolised via the Krebs cycle. The reaction of PDHC is highly regulated as it is irreversible and discriminates between metabolites that can be used for gluconeogenesis and others that can only be used for aerobic oxidation. Defects in PDHC can be due to mutations either in its subunits or in its regulatory kinases and phosphatases. Furthermore, the reaction depends on five different cofactors: thiamine pyrophosphate (TPP), protein-bound lipoic acid (lipoate), flavin adenine dinucleotide (FAD), coenzyme A and nicotinamide adenine dinucleotide (NAD^+^).

We realised that a couple of our patients with defects in the pyruvate oxidation route did not show any mutations when we sequenced all PDHC subunits and also the regulatory phosphatases and kinases. Finally, we suspected that also defects in one of the involved cofactors might result in a pyruvate oxidation defect. We started to investigate the involved cofactors TPP and lipoate, which resulted in the identification of two new genetic defects: thiamine pyrophosphokinase (TPK1) deficiency (6) and lipoate synthase (LIAS) deficiency (7).

TPK1 is necessary for the formation of TPP from thiamine (vitamin B1). Thiamine is taken up from the diet and transported to the cells including the two solute carriers SLC19A2 and SLC19A3. In the cytosol of cells, thiamine is pyrophosphorylated in a single step by TPK1 using ATP and thiamine as substrates and magnesium ion as a cofactor. TPP is a cofactor for at least six biochemical reactions in the cell (four mitochondrial, one cytosolic and one peroxisomal) and is transported into mitochondria by a specific carrier SLC25A19. Patients with TPK1 deficiency usually present with early-onset, recurrent encephalopathic episodes, frequently triggered by infection, followed by ataxia, epilepsy, pyramidal signs and dystonia. Treatment with high doses of thiamine (100–500 mg/ kg body weight) resulted in clinical improvement or stabilisation in approximately half of the patients, maybe depending on the degree of residual TPK1 activity. Interestingly, the main symptoms of TPP deficiency seem to be due to reduced pyruvate dehydrogenase and 2-oxoglutarate dehydrogenase, as can be inferred from elevated lactate and 2-oxoglutarate. The deficiency of the other four enzymes seems to have a minor contribution to the disease phenotype.

LIAS is required for the synthesis of the lipoate cofactor, which takes place on the octanoyl-moiety bound to a specific lysine residue at the H protein (GCDH) of the glycine cleavage system in mitochondria. This octanoyl-moiety is provided by the mitochondrial fatty acid synthesis, a metabolic pathway of bacterial ancestry. LIAS is an iron-sulphur protein containing two [4Fe-4S] clusters. S-Adenosyl-L-methionine (SAM) is a substrate and sulphur donor of this complicated biochemical reaction. Defects in lipoate formation due to LIAS deficiency affect 2-keto acid dehydrogenases like pyruvate dehydrogenase and 2-oxoglutarate dehydrogenase and also the glycine cleavage system. Elevated glycine and lactate, as well as 2-oxoglutarate levels, are therefore a typical signature of LIAS deficiency. Clinically, patients with LIAS deficiency can present in the first days of life with severe epileptic seizures resembling patients with classical non-ketotic hyperglycinaemia (NKH).

## From Gene to Function

Though all modes of inheritance have to be considered in disorders of the mitochondrial energy metabolism, the vast majority of defects is inherited in an autosomal recessive way. Consanguineous families, ideally with several affected individuals, have been the basis for narrowing down homozygous regions in their genomes and thus reducing the number of potentially novel candidate genes.

We have successfully used homozygosity mapping to identify *TMEM70* as the causative gene for patients with neonatal hypertrophic cardiomyopathy, lactic acidosis, 3-methylglutaconic aciduria, a decrease of ATP synthesis and a deficiency of the F_1_F_O_ ATP synthase (8). TMEM70 is a transmembrane protein, whose function was unknown before our linkage to ATP synthase deficiency. Homologues of TMEM70 can be found in animals and plants but not in fungi. As assembly intermediates were identified in the TMEM70 deficient cells, a function of TMEM70 in ATP synthase biogenesis has been concluded.

Another example of a decrease in ATP synthesis in muscle tissue is Sengers syndrome. Patients with Sengers syndrome usually present with congenital cataract, hypertrophic cardiomyopathy, muscle weakness and lactic acidosis. The amount and activity of the F_1_F_O_ ATP synthase were found to be normal, as was the mitochondrial phosphate carrier. Another part of the ATP synthesis machinery, the adenine nucleotide translocator (ANT), was shown to be reduced (9). However, sequencing of all tissue-specific isoforms of ANT did not reveal any abnormalities, leaving the precise pathomechanism enigmatic. Finally, exome sequencing revealed combined heterozygous stop variants in a gene called *AGK* in a patient with severe Sengers syndrome, and further biallelic *AGK* variants were identified in other patients with Sengers syndrome (10). AGK encodes an acylglycerol kinase, which might be involved in the mitochondrial phospholipid metabolism and could be linked to ANT via the mitochondria-specific phospholipid cardiolipin. However, recent papers showed the role of AGK in mitochondrial protein import, which, on the other hand, does not answer the tissue-specific phenotype of biochemical abnormalities in Sengers syndrome. Further studies are therefore required to understand the physiologic function of AGK.

## ‘Genetics First’ – the NGS Era

**Figure 3:**
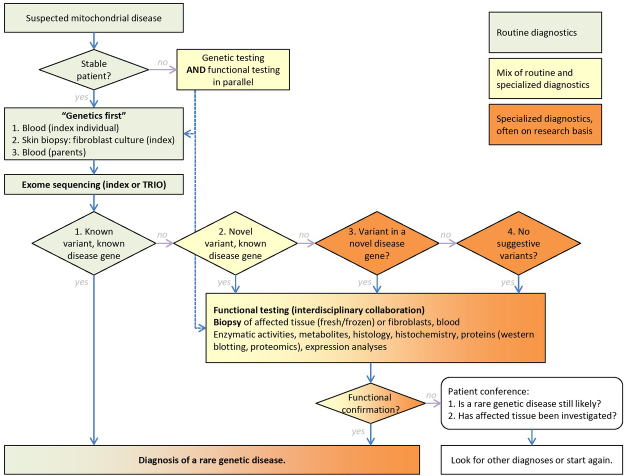
Diagnostic approach (flow chart) in the ‘genetics first’ era

In the second decade of the twentieth century, now in our fourth decade since 1980, due to the decreasing costs and improved technology in NGS techniques, Whole Exome Sequencing (WES) has conquered the field and is changing the diagnostic algorithm (Figure 3). First, the genetic first approach is attractive from the patient side, no biopsies are needed primarily, and furthermore in many diseases, where biochemically no clear interpretation or diagnosis was possible, the diseases could now be pinpointed at the genetic level. The diagnostic yield of WES in patients with suspected mitochondrial diseases is high, usually 40% or more, and, therefore, WES has to be recommended as first-line diagnostics, especially if the patient is in a stable clinical condition (11). Functional tests should be used as confirmatory investigations in a targeted way (e.g. enzymatic investigations, specific metabolite analysis, protein quantification and RNA expression) (12). Changing the order of investigations and implementing WES in the diagnostic algorithm substantially reduce the diagnostic odyssey, which was often the case for patients with such a heterogeneous disease.

**Figure 4:**
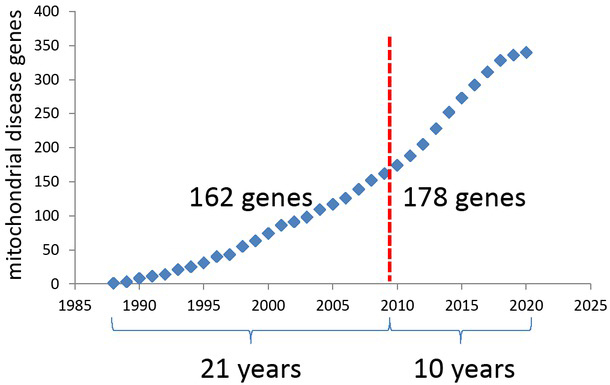
Timeline of the cumulative number of mitochondrial disease gene versus the year of the first report. (A) classical approach 1988–2009 and (B) genetics first approach 2010–2020 (March)

Although there was an increase in revealing novel disease-relevant genes (162 in 21 years, 178 in the last 10 years) (Figure 4), many challenges have emerged with this new diagnostic strategy.

**Figure 5:**
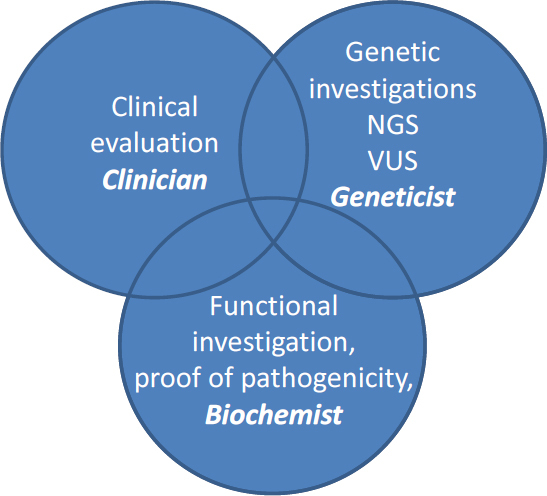
Clinical, genetic and functional competence with the interface between diagnostic and research are a prerequisite of diagnostic centres using the ‘genetics first’ approach

The indication for including diseases with possible mitochondrial pathophysiology for genetic diagnostics has become broader. Many clinical symptoms of mitochondrial diseases are unspecific, wide-ranging and very heterogeneous, and therefore, a broad overlap exists with other neurometabolic disorders. Furthermore, due to the minimal invasive character of the genetic first approach, an increasing number of patients with a suspected mitochondrial disease and milder or non-specific symptoms are included. Besides the classical known mitochondrial diseases, this approach enables the identification of new mitochondrial disease entities (13, 14, 15). In addition, also genetic diseases not related to the mitochondrial energy metabolism but with a clinical overlap to mitochondrial disorders can be identified. With this genetics first diagnostic approach, many patients remain unclear, e.g. with variants of unknown significance (VUS) in a known gene relevant for the mitochondrial energy metabolism or variants in a novel candidate gene. The confirmation by functional biochemical investigations (12) is a prerequisite to substantiate an underlying defect. It has been emphasised that also in the genetics first era a careful clinical characterisation is the basis for the subsequent investigations. It is our experience that the close interaction between the clinician, geneticist and biochemist helps enormously, especially in those complicated constellations, and increases the diagnostic yield (Figure 5).

## Future Aspects: Lessons We Have to Learn

The new genetic era has ofered a clear new challenge for the responsibility of the diagnostic centres: (1) The clinical condition of the patient has to be elucidated carefully, (2) the candidate genes coming from NGS have to be narrowed down based on this detailed knowledge of clinical features to pick out appropriate candidate genes and (3) VUS requires functional validation for confirmation of pathogenicity. Therefore, the interplay of the clinician, geneticist and functional biochemist is a prerequisite for a validated diagnosis, which has to be established in competence centres with high frequency and experience and exchange of knowledge with other comparable units and an interface between diagnostics and research.
